# Genomic Discoveries and Personalized Medicine in Neurological Diseases

**DOI:** 10.3390/pharmaceutics7040542

**Published:** 2015-12-07

**Authors:** Li Zhang, Huixiao Hong

**Affiliations:** 1 U.S. Food and Drug Administration, Center for Drug Evaluation and Research, 10903 New Hampshire Ave, Silver Spring, MD 20993, USA; 2 U.S. Food and Drug Administration, National Center for Toxicological Research, 3900 NCTR Road, Jefferson, AR 72079, USA; E-Mail: Huixiao.Hong@fda.hhs.gov

**Keywords:** genomics, personalized medicine, neurological disease

## Abstract

In the past decades, we have witnessed dramatic changes in clinical diagnoses and treatments due to the revolutions of genomics and personalized medicine. Undoubtedly we also met many challenges when we use those advanced technologies in drug discovery and development. In this review, we describe when genomic information is applied in personal healthcare in general. We illustrate some case examples of genomic discoveries and promising personalized medicine applications in the area of neurological disease particular. Available data suggest that individual genomics can be applied to better treat patients in the near future.

## 1. Introduction

The field of genetics is the study of genes, inheritance and genetic variation, while genomics is the study of the complete DNA sequence in the genome. In recent years, genomics gave birth to a series of other omics that refer to the complete collection of gene derivatives such as proteins, transcripts, or metabolites. Therefore, the broader and more inclusive term “genomics” sometimes refers to all large-scale approaches that are included in “omics”.

Personalized medicine refers to the use of individual unique genomic information to optimize patient care. Because, in each individual, the nature of the disease, the onset, the prognosis, and the drug response are different, the effective application of genomic findings to clinical practice becomes our predominant goal.

**Figure 1:**
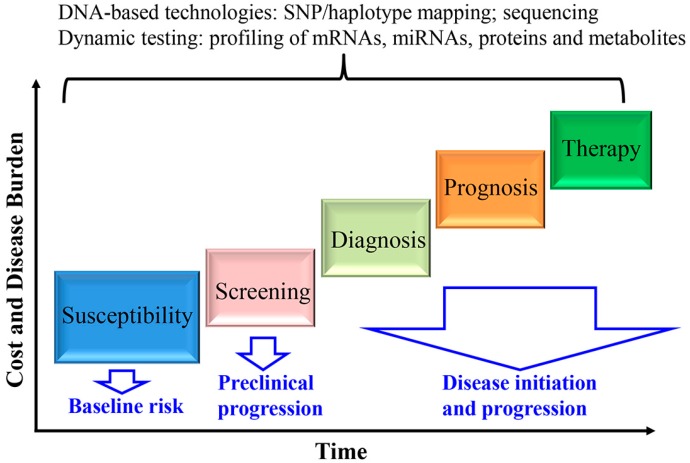
Applications of the human genome to personal healthcare.

In this review, we describe the important roles of genomic information applied in personal healthcare in general (Figure 1). First, disease susceptibility and risk can be identified at birth using DNA-based technologies, such as SNP genotyping, haplotype mapping or gene sequencing. Second, dynamic testing, including the profiles of mRNAs and microRNAs, and proteins and metabolites, combined with molecular imaging modalities may provide more precise means to access the risk of individuals during early initiating events of the disease. That information can also improve disease diagnosis and predict the disease progression. Third, when the decision is made to treat a condition, the selection of a therapy can be directed by the patient’s genetic makeup as well as by the understanding of the disease’s mechanism.

### 1.1. DNA-Based Technologies

Human genome variation is represented by single-nucleotide polymorphisms (SNPs), copy-number variations (CNVs), insertions and deletions. During the drug discovery phase, information about genetic variations is used to identify signaling pathways as therapeutic targets. This early-stage research tries to understand the disparity between patients and control populations. Genome-wide association study (GWAS) is an efficient method that detects human genome variations and reveals novel genes contributing to disease pathogenesis. Some known drug targets and associated pathways have been identified on the list of GWAS hits. In the NIH policy white paper (2011) [1], 44 genetic variants have been found to be strongly linked to type 2 diabetes susceptibility. Among them, six genetic variants are the primary drug targets while there are eight genetic variants associated with cellular, pharmacokinetic, pharmacodynamic, or clinical variations which respond to one or multiple drugs currently on the market. The other 30 variants on the list will further provide new insights into the underlying biology, disease mechanisms, and potentially novel therapeutic approaches.

As we understand, in reality, sometimes the contributions of genetic variants to the phenotypes are very small. The inconsistency in genotypic measurements diminishes the accuracy of genotypes and causes Type II errors in GWAS [2,3]. There are diverse sources of genotype inconsistency from SNP microarray technologies that affect the findings of GWAS, including genotyping technologies [4], the batch effect [5,6] and genotype calling algorithms [7,8,9,10]. Currently, next-generation sequencing (NGS) technologies have emerged as the most promising tools in genetic studies [11].

Advances in sequencing technology have reduced the costs to the point where a human genome can now be sequenced at the $1000 level [12]. Therefore, sequencing a patient’s genome will be part of standard medical testing in the future. NGS has now been used to sequence hundreds of human genomes and is being applied to identify the disease-related genes, which further helps to make a definitive diagnosis and even guide the treatment. Nicholas Volker’s case is a promising example [13]. He is the first patient diagnosed with an *XIAP* mutation and was saved by sequencing technology. In responding to President Obama’s announcement of a new initiative for precision medicine, the NIH is going to sequence one million human personal genomes. With the development of quality control metrics and standardization for NGS technologies and data analysis [14,15], NGS will accelerate the implementation of personalized medicine to improve public healthcare.

### 1.2. Dynamic Testing

Dynamic testing is a method to look at an individual’s molecular profile and study the genetic characteristics such as the messenger RNAs (mRNA), MicroRNAs, proteins and metabolites.

**Figure 2:**
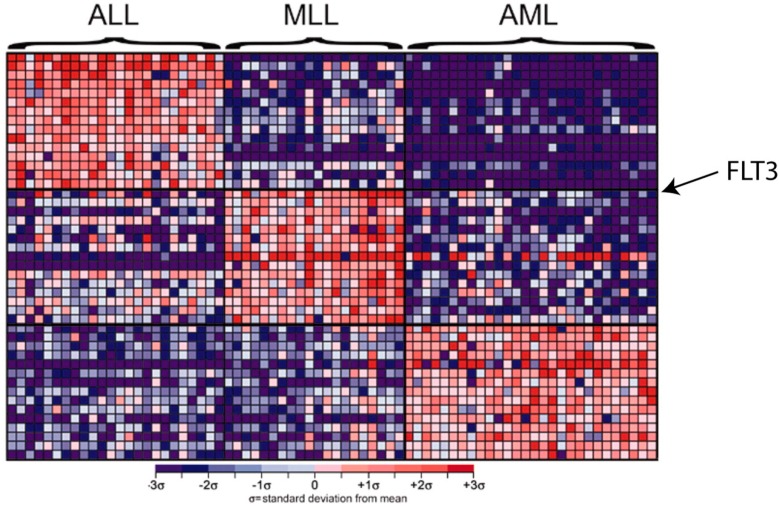
Diagnosis and disease classification using molecular profiles.

The unique pattern of molecular profiling can be used to explain disease heterogeneity and further classify the diseases. Figure 2 demonstrates the utilization of gene expression profiles of bone marrow samples to diagnose patients with hematologic malignancies [16]. Each column represents a bone marrow sample and each row corresponds to a gene. Shades of red indicate elevated expression while shades of blue indicate decreased expression. *FLT3* is the gene that is found to correlate most highly with the mixed-lineage leukemia (MLL) subtype. Several hundred studies have identified gene or protein expression profiles that predict clinical outcome and disease recurrence risks. Some are being routinely used for disease diagnosis and approved by the US FDA [17].

### 1.3. Pharmacogenomics

Pharmacogenomics refers to the use of complex molecular information from the genome to optimize drug dose or predict drug response, which is a valuable tool in the development of new drugs as well as in improving overall outcomes with current drugs. Hundreds of drug labels have included exposure information and clinical response variability in individuals with certain genotypes [18]. Among those drugs, Trastuzumab, a monoclonal antibody that interferes with *HER2*, is an example of a biologic therapy for which the diagnostic assay was developed to identify certain cancer patients who will most likely benefit. Trastuzumab was approved for about 10% of *HER2*-overexpressing patients. Due to the small sample size of the subgroup of patients, without a sensitive and accurate diagnostic assay, the drug might fail at the clinical trial stage. Clopidogrel is an antiplatelet agent used to prevent heart attack and inhibit blood clots in coronary artery disease. *CYP2C19* is an important drug-metabolizing enzyme. In 2010, the FDA put a black box warning on Clopidogrel to make patients and healthcare providers aware that patients with low activity of *CYP2C19*, representing up to 14% of patients, are at high risk of treatment failure and it is recommended that genetic testing is used to screen out those patients. Tetrabenazine is approved to treat Huntington's disease (HD) and is primarily metabolized by *CYP2D6*. Due to safety concerns caused by high exposure, patients with low activity of *CYP2D6* should be treated with lower doses compared with extensive and intermediate metabolizers.

### 1.4. Challenges of Using Genomics in Drug Development

It generally takes 10–15 years for an experimental drug to go from the lab to US patient use. Only five in 5000 compounds at preclinical testing can enter the phase I stage. Additionally, only one of those five is approved for the market. On average, it costs a company $1.2 billion. Based on the awareness of the drug development and approval process, researchers who are interested in translating genomic discoveries into personalized drug therapy should understand the challenges involved. (1) Drug development is extremely risky. Every year, about 10% of drug candidates can pass preclinical development to get approval. In recent years, almost half of the potential therapeutics failed with late-stage failures. Moreover, pharmaceutical product candidates receive extensive government regulations. Those factors lead to a conservative attitude toward using genomic approaches in pharmaceutical development. (2) Since the cost for the development of an innovative drug is very high and the time to create a successful drug is extremely long, pharmaceutical companies are reluctant to use genomic approaches to limit the indicated patient population unless “one-size-fits-all” development fails. (3) Even after drug approval, the manufacturer might not be able to make much profit by continuing genomic post-marketing studies due to patents’ limited lifespans.

There are additional challenges for genomics to be applied in neurological drug development. (1) There is structural and functional heterogeneity of the individual brain. (2) There are difficulties for acquiring quality data from specimens for reliable analysis. Additionally, developing a human brain model is not easy and animal models cannot display the full range of human phenotypes. (3) Neurological evaluations for disease severity or progression are always subjective due to patients’ or investigators’ reports. Better phenotypes are needed to categorize neurological diseases based on biology and etiology.

## 2. Genomic Discoveries and Personalized Medicine Applications in Neurological Diseases

Advances in genome technologies and the recent huge outpouring of genomic information related to neurological diseases have accelerated the convergence of discovery science and clinical medicine. In this section, we summarize some rare disease and complex disease case examples which may have the potential to translate genomics into therapeutics and make personalized medicine possible. Table 1 lists the disease, disease type, susceptibility genes, clinical utility, and available therapy.

**Table 1:** Case example summarization.

Disease	Disease Type	Susceptibility Genes	Clinical Utility	Available Therapy
Duchenne Muscular Dystrophy	Rare	*DMD*	disease-causing gene, drug target	Corticosteroids to control the symptoms
Early-onset familial Alzheimer’s Disease	Complex	*APP, PSEN1, PSEN2*	disease-causing gene	Tacrine, Rivastigmine, Galantamine, Donepezil and Memantine to control the symptoms
Alzheimer’s Disease	Complex	*APOE, CR1, BIN1, CLU, MS4A6A, PICALM, ABCA7, CD33, PTK2B, SORL1, MEF2C, ZCWPW1*, and *CASS4*	increase disease risk	
		*BACE1*	increase disease risk, drug target	
Parkinson’s Disease	Complex	*LRRK2, PARK2, PARK7, PINK1, and SNCA*	disease-causing gene, drug target	Levodopa, dopamine agonists, and MAO-B inhibitors to control the symptoms
		*MAO-B*	increase disease risk, drug target	
		*GBA* and *UCHL1*, *etc.*	increase disease risk	
Epilepsy	Complex	*SCN1A* and *PCDH7*	increase disease risk	Carbamazepine, Oxcarbazepine and Phenytoin to control the symptoms
		HLA-B*1502 and CYP2C9	drug safety gene	

### 2.1. Duchenne Muscular Dystrophy (DMD)

DMD is a recessive X-linked disease which affects around one in 3600 males, and females rarely exhibit signs of this disease. This disorder is caused by a mutation in the *DMD* gene on the X chromosome such that it no longer produces functional dystrophin protein. Dystrophin is an important structural component within skeletal and cardiac muscle tissue. A deficiency of dystrophin will result in muscle degeneration and premature death. Most patients are wheelchair-dependent by age 12 and the average life expectancy is around 25. There is no cure for DMD, and an ongoing medical need has been recognized by regulatory authorities. Current treatments including corticosteroids are mainly used to control the onset of symptoms [19]. The *DMD* gene consists of 79 exons. A deletion of exon 71 would be considered “in frame” because exons 70 and 72 could still be spliced to allow transcription. However, deletion of exons 48 through 50 would be “out of frame” since 47 and 51 do not splice back together. Recently, a number of experimental therapeutic approaches are being developed that aim to restore the absent dystrophin protein in the muscles. The treatment strategy is to skip the exon to restore a genetic “reading frame”. The functional mRNA is made by splicing out the introns in the pre-mRNA of the dystrophin gene. However, a deletion of exon 50 in the dystrophin gene disrupts the mRNA reading frame and results in the interruption of dystrophin protein production. Antisense knocks out exon 51. The rest of the exons join up together, resulting in a shortened mRNA reading frame but functional dystrophin proteins. If the compounds work, a muscle biopsy should show increased dystrophin expression and patients would remain ambulatory as measured using a six-minute walking distance. Further information can be referred to in DMD draft guidance [20]. The dystrophin gene is very large and the genetic errors associated with DMD occur in multiple locations. The clinical trials focusing on exon 51 skipping hope to help about 13% of patients with DMD. Such a patching and alternating strategy can certainly be carried out in other rare genetic diseases based on the thorough understanding of the disease mechanism.

### 2.2. Alzheimer’s Disease (AD)

Alzheimer’s disease is a degenerative brain disease and it is ultimately fatal. Currently, 5.3 million Americans are living with AD. Causes of AD have not been fully understood and genetics may play an important role in disease development. Early-onset familial AD (EOFAD) is a hereditary condition that accounts for up to 5% of all AD patients. The genetic linkage analysis revealed that 1% of AD patients have the disease due to mutations in the amyloid precursor protein (*APP*) and in the genes for the presenilin 1 and presenilin 2 proteins. Although these three genes reside on different chromosomes, they belong to the same biochemical pathway, which alters the protein production of Aβ and causes neuron death. Individuals with mutations in any of the three genes tend to develop AD before age 65, even as early as in their 30s, while the majority of individuals with AD have late-onset disease, which occurs at age 65 or later. In contrast to EOFAD, risk genes for late-onset AD display a complex interaction pattern that involves not only the genes but also environmental factors. The best known genetic risk factor is the inheritance of the ε4 allele of the apolipoprotein E (*APOE*). Between 40% and 80% of people with AD possess at least one *APOE* ε4 allele [21]. However, unlike the three mutations in the early-onset AD patients, the *APOE* ε4 allele is neither necessary nor sufficient for causing AD. The current hypothesis is that *APOE* ε4 increases a carrier’s risk for hypercholesterolemia, which leads to elevate the accumulation of Aβ. The completion of GWAS has discovered other potential new susceptibility genes during recent years. Lamber *et al.* [22] found 19 loci that reached genome-wide significance (*p* < 5 × 10^−8^) with meta-analysis of 74,046 individuals, 11 of which are newly associated with AD. Beecham *et al.* [23] confirmed 12 known non-*APOE* genetic risk loci (*CR1, BIN1, CLU, MS4A6A, PICALM, ABCA7, CD33, PTK2B, SORL1, MEF2C, ZCWPW1*, and *CASS4*) were associated with clinically defined AD dementia. Jonsson *et al.* [24] identified A673T in the *APP* gene that protects against AD and cognitive decline in the elderly without AD. *In vitro* results showed that the substitution reduced approximately 40% of the formation of amyloidogenic peptides, which further support the involvement of Aβ peptides in AD pathology and *BACE1* as a drug target. Five medications are currently used to treat the cognitive problems of AD: four are cholinesterase inhibitors (tacrine, rivastigmine, galantamine, and donepezil) and the other (memantine) is an *N*-methyl-d-aspartate receptor antagonist [25], and they are all symptomatic treatments. Since 2003, no new treatment has been approved and no medication has been clearly shown to slow or stop the progression of AD. There is an urgent need to find new therapies for AD. Pharmaceutical Research and Manufacturers of America published a report in 2013 [26] which included 64 medicines being developed by companies conducting trials in the United States and abroad. Almost half of the drugs targeted the amyloid-beta protein. In only a few years, more progress has been made in AD genetics than the past several decades. This is due to the increasing application of genome-wide screenings in the quest for novel disease genes. As the science advances, regulatory positions will evolve as well. In general, regulatory criteria for the marketing of therapies for AD require the demonstration of cognitive efficacy and improvements in function [27]. The genomics discoveries will eventually provide more solid support for the knowledge to be translated into targeted therapies and clinical applications.

### 2.3. Parkinson’s Disease (PD)

PD is one of the most common neurological disorders, affecting around 1% of the population over the age of 65. The motor symptoms of Parkinson’s disease, such as shaking, rigidity, and difficulty with walking and gait, may result from the death of dopamine-generating cells. PD was not considered a genetic disorder; however, around 15% of individuals with PD have a family history of the disease [28]. Familial cases of PD can be caused by mutations in the *LRRK2, PARK2, PARK7, PINK1*, or *SNCA* genes, or by alterations in genes that have not been identified. Mutations in some of these genes may also play a role in cases that appear to be sporadic (not inherited) [29]. Among those genes, *LRRK2* and *SNCA* show an autosomal dominant inheritance pattern while *PARK2*, *PARK7* and *PINK1* show an autosomal recessive inheritance pattern. Alterations in certain genes, including *GBA* and *UCHL1*, do not cause PD but appear to increase the risk of developing the condition in some families. GWAS was used to identify and replicate susceptibility factors for PD. Recently Nalls *et al.* [30] conducted a meta-analysis of Parkinson’s disease genome-wide association studies using a common set of 7,893,274 variants across 19,081 cases and 100,833 controls. They identified and replicated 28 independent risk variants for Parkinson’s disease across 24 loci. In addition, some groups have started genome sequencing as well as metabolomics and proteomics in PD patients. These studies have both affirmed the central role of genes previously linked to PD and implicated new targets/pathways that can be explored in drug development.

There is no cure for PD. The main families of drugs useful for treating motor symptoms are levodopa, dopamine agonists, and *MAO-B* inhibitors [31]. Levodopa has been the most widely used treatment for more than 30 years. Levodopa can be converted into dopamine in the neurons; therefore, it is able to temporarily diminish the motor symptoms [31]. Dopamine agonists include bromocriptine, pergolide, pramipexole, ropinirole, piribedil, cabergoline, apomorphine and lisuride. They bind to dopaminergic post-synaptic receptors in the brain. They are the preferred initial treatment for earlier onset, as opposed to levodopa in later onset, due to a lesser effectiveness in controlling symptoms [28]. *MAO-B* inhibitors (selegiline and rasagiline) increase the level of dopamine in the basal ganglia by blocking monoamine oxidase B enzyme activities [31]. *MAO-B* inhibitors are used as a monotherapy to improve motor symptoms and reduce fluctuations between on and off periods. Like dopamine agonists, they can delay the need for levodopa in early disease, but they produce more side effects and are less effective than levodopa [31]. Some other drugs such as anticholinergics may be useful as a second-line treatment. Disease-modifying therapies are designed to prevent and slow the progression of PD. They target different proteins and pathways known to play a role in the disease. As we discussed, *SNCA*-encoding alpha-synuclein has been confirmed to be associated with PD. Studies found alpha-synuclein protein clumps in the brains of PD patients with toxic effects. Therefore, Affitope is a vaccine that stimulates antibodies to reduce the aggregation of alpha-synuclein in the brain and Phenylbutyrate is being developed for alpha-synuclein clearance from the brain as well [32]. Both trials are at the phase I stage.

### 2.4. Epilepsy

Epilepsy is a neurological disorder in which nerve cell activity in the brain becomes disrupted, causing seizures or periods of unusual behavior, sensations and, sometimes, loss of consciousness [33]. About 1% of people worldwide have epilepsy [34]. Epilepsy is not a single condition. There are multiple causes of brain dysfunction that lead to seizures. Genetics are believed to be involved in the majority of epilepsy cases [35]. Some epilepsies are caused by a single gene defect (1%–2%); the majority of them are due to the interaction of multiple genes and environmental factors. Therefore, heterogeneity extends to many aspects of epilepsy. Causation, susceptibility, clinical symptoms, treatment response, and adverse reaction are all variable. These observations have motivated an unbiased genome-wide approach to search for common variants that might cause or contribute to epilepsy. One recently published study based on 8696 cases and 26,157 controls [36] identified that *SCN1A* and *PCDH7* are associated with epilepsy. In routine clinical practice, the variation in treatment response is the most commonly encountered unpredictability and it is possible that individual genomic variation contributes to these phenotypic drug response variations. There are over 20 anti-epileptic drugs (AEDs) and research has validated two genomic associations for AED usage. Individuals who have HLA-B*1502 are more likely to experience a severe skin disorder called Stevens-Johnson syndrome in response to carbamazepine, oxcarbazepine and phenytoin [37]. Genetic testing for this allele in people with Asian ancestry is now recommended prior to using these drugs. The second association is for phenytoin metabolism and rare variants on the gene encoding the major enzyme *CYP2C9*. The *2 and *3 alleles are associated with decreased metabolism and potential toxicity in North American patients. Therefore, genetic testing is recommended to adjust doses of phenytoin. For patients with low activity of *CYP2C9*, at least a 50% reduction of the starting dose is recommended, with subsequent maintenance doses adjusted based on response.

## 3. The Outlook for Personalized Medicine in Neurological Diseases

Dozens of genes have been found to be associated with various neurological diseases. Our understanding of the genetic etiology will change over time along with the advancement of technologies. Although genomics knowledge is still limited, many clinical institutes have considered to routinely use it in the selection of the ideal treatment for an individual patient. The goal in neurological personalized medicine is to improve currently existing treatments and accelerate future drug therapies. Disease-associated biomarkers will likely advance the translation of scientific discoveries into clinical applications. In addition to behavior and imaging endpoints, some genomics biomarkers can also be used to predict response and follow pathology. The development of disease classifiers could quantify the changes in disease status to improve clinical management and research. The Critical Path Institute has initiated a “Coalition Against Major Diseases (CAMD)” [38] program and made efforts to integrate data from controls in industry clinical trials. Some sites are also attempting to combine existing genomics data for better statistical analysis. However, because DNA collection is always voluntary, a global collaboration is needed to have more study participants and sample collections.

The genomics discoveries greatly help us to understand disease mechanisms, which lead to the identification of druggable targets once the mutations are found. However, the bottlenecks along the path of moving neurological disease treatments from the bench to the bedside are numerous and formidable compared with other therapeutic areas. The current missing gaps may be due to the following. (1) Most neurological diseases are complex diseases with lots of gene-lifestyle interactions involved, and they may not have a simple, unique genetic solution. (2) Neurological diseases are chronic and irreversible, which requires long-term disease control and management. (3) The disease-modifying treatment development cost is extremely expensive and double-blind clinical trials generally need 18 months or longer to show treatment effects on slowing disease progression. As highlighted in this paper, what we are certain of is that the ability to understand the clinical features of a patient’s genetic profile and the knowledge of disease mechanisms will facilitate new targeted therapies. As President Obama pointed out in the Precision Medicine Initiative, it calls for continuous efforts to transform information into knowledge which helps neurological patients receive better treatments by using their individual genome characteristics.
